# Supplementary material for: Collaborative optimization of multi-modal transport solutions for urban-rural bus routes
Source: PLoS One. 2024 Oct 8;19(10):e0309096. doi: 10.1371/journal.pone.0309096 (PMC11460678; doi:10.1371/journal.pone.0309096)
Supplement: S1 File — Original files of investigated data and algorithm codes. (ZIP) [file pone.0309096.s001.zip › raw dataset/7-Lingo codes.docx]

**(1) Codes for optimizing the passenger travel time cost**

| Sets definition | sets:  VAR1/1,2,3,4,5,6,7,8,9,10,11,12,13,14,15,16/:u;  VAR2/1,2,3/:m;  row/1,2,3,4,5,6,7,8,9,10,11,12,13,14,15,16/;  col/1,2,3,4,5,6,7,8,9,10,11,12,13,14,15,16/;  links(row,c5ol):q,L;  proportion2/1,2/:p2;  DUM1/1,2,3,4,5,6,7,8/:DC1;  DUM2/1,2,3,4,5/:DC2;  DUM3/1,2/:DC3;  endsets |
| --- | --- |
| Data input | DATA:  q =@OLE('D:\客流OD矩阵.xlsx','OD');  L =@OLE('D:\起讫点距离矩阵.xlsx','距离数据');  p2=0.35,0.13;  p3=0.46;  t1=28;  v1=25;  v2=35;  v3=30;  cop=0.2;  vot=0.3;  b1,b2,b3=50,35,20;  u=1,,,,,,,,,,,,,,,1;  DC1=50,75,117,133,142,157,182,177;  DC2=217,223,247,239,210;  DC3=83,40;  ENDDATA |
| Objective function | sumw1=@sum(row(i)\|i#le#8:@sum(col(j)\|j#ge#i:q(i,j)*  ((1-u(i)*p2(1))*30/m(1)+u(i)*p2(1)*30/m(2))));  sumw2=@sum(row(i)\|i#eq#14#and#i#eq#15:@sum(col(j)\|j#ge#i:q(i,j)*  ((1-u(i)*p2(1))*30/m(1)+u(i)*p2(1)*30/m(2))));  sumw3=@sum(row(i)\|i#ge#9#and#i#le#13:@sum(col(j)\|j#ge#10#and#j#le#14:q(i,j)*((1-u(i)*p2(2)-p3)*30/m(1)+u(i)*p2(2)*30/m(2)+p3*30/m(3))));  Twait=sumw1+sumw2+sumw3;  Tin1=@sum(row(i)\|i#ge#1#and#i#le#8:@sum(col(j)\|j#ge#i:q(i,j)*  ((1-u(i)*p2(1))*(60*L(i,j)/V1+@sum(row(s)\|s#ge#i+1#and#s#le#j:t1/60))+u(i)*p2(1)*(60*L(i,j)/V2+@sum(row(s)\|s#ge#i+1#and#s#le#j:u(s)*t1/60)))));  Tin2=@sum(row(i)\|i#eq#14:@sum(col(j)\|j#ge#i:q(i,j)*((1-u(i)*p2(1))*(60*L(i,j)/V1+@sum(row(s)\|s#ge#i+1#and#s#le#j:t1/60))+u(i)*p2(1)*(60*L(i,j)/V2+  @sum(row(s)\|s#ge#i+1#and#s#le#j:u(s)*t1/60)))));  Tin3=@sum(row(i)\|i#eq#15:@sum(col(j)\|j#ge#i:q(i,j)*((1-u(i)*p2(1))*  (60*L(i,j)/V1 +t1/60)+u(i)*p2(1)*(60*L(15,j)/V2+t1/60))));  Tin4=@sum(row(i)\|i#ge#9#and#i#le#13:@sum(col(j)\|j#ge#10#and#j#le#14:q(i,j)*((1-u(i)*p2(2)-p3)*(60*L(i,j)/V1+@sum(row(s)\|s#ge#i+1#and#s#le#j:t1/60))+  u(i)*p2(2)*(60*L(i,j)/V2+@sum(row(s)\|s#ge#i+1#and#s#le#j:u(s)*t1/60))+p3*(60*L(i,j)/V2+@sum(row(s)\|s#ge#i+1#and#s#le#j:t1/60)))));  Tin=Tin1+Tin2+Tin3+Tin4;  Cpe=cop*(@sum(row(i):@sum(col(j)\|j#ge#i:q(i,j)*(1-u(i))*p2(1)*  (60*L(i,j)/V1-60*L(i,j)/V2)*vot)));  min=(Twait+Tin)*vot+Cpe; |
| Model constraints | @for(VAR1:@bin(u));  @for(VAR2:@gin(m));  @for(VAR2(i):m(i)<6);  @for(VAR2(i):m(i)*b3<247);  @for(DUM1(k):b1*m(1)+b2*u(k)*m(2)>=DC1(k));  @for(DUM2(k):b1*m(1)+b2*u(k+8)*m(2)+b3*m(3)>=DC2(k));  @for(VAR1(i)\|i#eq#14:b1*m(1)+b2*u(i)*m(2)>=DC3(1));  @for(VAR1(i)\|i#eq#15:b1*m(1)+b2*u(i)*m(2)>=DC3(2));  costvalue=@if(@sum(VAR1(i):u(i))#ge#3,1,0);  m(2)>=costvalue;  @sum(VAR1(i)\|i#ge#2#and#i#le#15:u(i))<=6;  @for(DUM2(k):0.6*(b1*m(1)+b2*u(k+8)*m(2)+b3*m(3))<=DC2(k)); |

**(2) Codes for optimizing vehicle operating time cost**

| Sets definition | sets:  VAR/1,2,3,4,5,6,7,8,9,10,11,12,13,14,15,16/:u;  FLEET/1,2,3/:m,coe;  DUM1/1,2,3,4,5,6,7,8/:DC1;  DUM2/1,2,3,4,5/:DC2;  DUM3/1,2/:DC3;  endsets |
| --- | --- |
| Data input | data:  coe=56.1,51.7,29.5;  L1=23.7733;  L2=9.6015;  t1=28;  V1=25;  V2=35;  V3=30;  b1=50;  b2=35;  b3=20;  u=1,,,,,,,,,,,,,,,1;  DC1=50,75,117,133,142,157,182,177;  DC2=217,223,247,239,210;  DC3=83,40;  enddata |
| Objective function | min=coe(1)*m(1)*(L1/V1+15*t1/3600)+coe(2)*m(2)*(L1/V2+  @sum(VAR(i):u(i)*t1/3600))+coe(3)*m(3)*(L2/V3+5*t1/3600); |

**(3) Codes for weighted objective programming**

| Data input | a=0.6;  b=0.4; % a+b=1  COS1min=3592.5;  COS2min=259.96; |
| --- | --- |
| Objective function | min=a*(COS1-COS1min)/0.8/COS1min+b*(COS2-COS2min)/2/COS2min； |
