# Supplementary material for: Collaborative optimization of multi-modal transport solutions for urban-rural bus routes
Source: PLoS One. 2024 Oct 8;19(10):e0309096. doi: 10.1371/journal.pone.0309096 (PMC11460678; doi:10.1371/journal.pone.0309096)
Supplement: Attachment — Submitted filename: Response letter 0605.doc [file pone.0309096.s002.doc]

**Original Manuscript ID:** PONE-D-24-09686

**Original Article Title: “**Collaborative Optimization of Multi-modal Transport Solutions for Urban-Rural Bus Routes”

**To:** PLOS ONE Editor

Dear Editor,

Thank you for giving us a chance to revise our paper, and thank you for your previous approvement of our request on revision deadline extension.

Over the past two months, we have spent great effort refining the manuscript, where the typing mistake, word usage, grammar error and sentence structure have been modified. Meanwhile, the comments raised by Reviewers and editors have got replied and implemented, together with the responses to Journal Requirements. Please see the separate response document uploaded in the system.

We are uploading (a) response document to the reviewers’ and editors’ comments (below), (b) a marked-up manuscript with indicated changes under the track changes mode in MS word, and (c) an unmarked manuscript without tracked changes.

Any further comments or requirements are welcome to us. Our deepest gratitude goes to you and the reviewers for your careful work. Thank you!

Best regards.

**Reviewer#1, Comment #1:** Please list the contributions of this paper.

**Author response:** Thanks for your comment!

**Author action:** In the revised conclusion section, contributions of this paper have been highlighted as:

(1) A simplified bi-objective programming model considering the traffic and trip characteristics along the urban-rural bus corridor. The simplifications are reflected in the objective formulation and parameter calibration. The objectives focus on the perceivable passenger travel time consumption and bus running cost, and the parameters are calibrated based on field observation and questionnaire investigation, in order to avoid the uncertain influences from modal service pricing, passenger preference, individual attribute, running delay and etc.

(2) An optimal scheme recommendation approach based on the sensitivity of sub-objective weights. Objective values of representative weight combinations have been discussed, together with the anticipated ticket incomes under given pricing systems, which can be provided as decision-making references for bus operators.

(3) A generalizable and executable model solving architecture. The paper presented a complete model solution method including the passenger trip characteristic analysis, the input parameter calibration, the modal service constraint formulation and the concrete code programming. Bus operators can quickly adjust the multi-modal operation scheme once the passenger trip distribution or vehicle service encounters significant fluctuations.

**Reviewer#1, Comment #2:** The presentation of this paper need to be improved.

**Author response:** Thanks for your comment, we have polished the manuscript by improving language and supplementing necessary descriptions, illustrations and tables. Please see the revised version.

**Reviewer#1, Comment #3:** The part of literature review need to be organized more clearly and logical. It’s not very well to summarize an article by a sentence.

**Author response:** Thanks for your valuable comment! For those literatures summarized by a sentence, necessary backgrounds, applications or advantages have been supplemented. Please see the revised manuscript.

**Reviewer#1, Comment #4:** In the section of “collaborative modelling”, authors proposed a complex optimization model with many assumptions and variables. Please illustrate a figure to show the framework of the proposed model. It’s difficult to understand the optimization method here.

**Author response:** Thanks for your comment.

**Author action:** In the revised manuscript, a framework of the proposed has been added in order to illustrate the proposed model, as shown in **Figure S1**. The newly added figure emphasizes the model formulation, which is different from the figure illustrating solving process in the “solution procedure” section. Meanwhile, please note that figures are uploaded separately in the submission system.

**Figure S1. The newly added modelling framework of proposed method**

**Reviewer#1, Comment #5:** In line 188 page 8, the variable of vot is used to calculate the time value from time cost. But the value is different in waiting, on-board or other situation in unit time. Please give explanation.

**Author response:** Thanks for your comment. *vot* is the abbreviation for "value of time", referring to the unit time cost per time period. In our study, the basic monetary unit of China is Yuan, and the time period is counted by minutes, therefore, the unit of *vot* is Yuan/(pax·min)..

**Author action:** In order to address the value of *vot* more adequately, necessary explanations have been supplemented in the parameter calibration in the revised manuscript.

Generally, *vot* varies with the income of passengers, for example, the *vot* of a farmer is usually lower than an engineer in China. Because it is hard to know the vocation of all passengers, the *vot* for urban-rural bus passengers is taken as 0.3 Yuan/(pax·min) upon a questionnaire analysis of 77 investigated passengers, represented sample distribution are listed in **Table S1**.

However, as you mentioned, the value of *vot* may differ in the waiting and on-board period, but it depends on the psychological status and perception of different passengers, which is currently hard for us to quantify. We will consider this valuable suggestion in our subsequent study on hybrid electrical and fuel buses timetabling. Thank you and hope for your understanding.

**Table S1. Represented passenger income distribution**

| Vocation | No. of samples | Proportion | Average income (Yuan/month) | Working hours  (hours/day) | *Vot*  (Yuan/min) | Average weighted *Vot* |
| --- | --- | --- | --- | --- | --- | --- |
| Farmer | 15 | 19.48% | 2300 | 6 | 0.21 | 0.297 |
| Worker | 21 | 27.27% | 5100 | 9 | 0.31 |  |
| Waitress | 12 | 15.58% | 4500 | 6.5 | 0.38 |  |
| Teacher | 3 | 3.90% | 6800 | 8 | 0.47 |  |
| Engineer | 4 | 5.19% | 6000 | 8 | 0.42 |  |
| Public servant | 2 | 2.60% | 5500 | 8 | 0.38 |  |
| Salesman | 7 | 9.09% | 5700 | 7.5 | 0.42 |  |
| Cleaner | 3 | 3.90% | 4300 | 6 | 0.39 |  |
| Security staff | 4 | 5.19% | 3800 | 12 | 0.18 |  |
| Student | 6 | 7.79% | 0 | 0 | 0 |  |

**Reviewer#1, Comment #6:** Public transportation network optimization should take the change of travel demands, road condition and other factors during the whole daytime into account. Here, some factors (such as road condition and so on) have a significant effect on time value calculation. Please consider.

**Author response:** Thanks for your comment. Necessary explanations have been added before model formulation, together with the newly added modelling framework mentioned in the response to your 4^th^ comment. (Reviewer #1, Comment #4).

**Author action:** The road traffic factors affecting the corridor bus operation usually include the road level, road traffic volume, signal control, public transport composition and passenger demand, where the signal control and passenger demand have been taken into consideration instead of the other factors, based on the following analysis:

(1) In the urban-rural area, roads usually include the express way, the arterial road, the collector road and the village road. Different road levels correspond to different lane number and speed distribution. The urban-rural corridors are usually fixed as arterial roads.

(2) Road traffic volume affects the level of congestion directly. However, the traffic volume of arterial roads along the urban-rural corridor are well below the designed road capacity. Therefore, the bus running speed will not get affected by road traffic volume.

(3) Bus running delay caused by signal control is inevitable. The average running speeds (travel speeds) of different bus service modals used in the model formulation are calculated considering the aggregate delay time at intersections based on real observed data. Necessary data are listed in the table below (**Table S2**). Note that the additional time consumption caused by stopping at one station (average stop delay) is 25 s.

(4) In urban areas, public transport composition has a significant influence on bus operation due to the competition relations. E.g., metro, tramcar and taxi will attract large number of passengers from regular bus system. However, the public transport along the urban-rural corridor is rather single, and mainly depends on the bus service.

(5) Passenger demands are necessary for the optimization of bus operation schemes. The station alighting & boarding passengers and the passenger volume exchange between stations are basic demand inputs of proposed optimization model.

**Table S2. Running speed observation of represented bus modals**

| Service model | Bus route | Observed running distance (km) | Observed time (min) | Intersection delay (min) | Stop delay (min) | | Running time (min) | Travel speed  (km/h) |
| --- | --- | --- | --- | --- | --- | --- | --- | --- |
|  |  |  |  |  | stops | time |  |  |
| Suttle bus | 15 | 12.1 | 30.09 | 2.81 | 7 | 2.92 | 24.36 | 29.8 |
|  | 15 special | 12.1 | 31.30 | 3.33 | 6 | 2.50 | 25.47 | 28.5 |
|  | 105(night) | 14.98 | 36.68 | 4.12 | 9 | 3.75 | 28.81 | 31.2 |
| Rapid bus | Youyang sightseeing | 19.22 | 41.09 | 6.15 | 5 | 2.08 | 32.85 | 35.1 |
|  | Songyang express | 17.54 | 37.69 | 5.52 | 4 | 1.67 | 30.50 | 34.5 |
| All-stop bus | 103 | 17.03 | 50.28 | 5.71 | 13 | 5.42 | 39.15 | 26.1 |
|  | Guoyang trunk | 11.65 | 33.87 | 3.02 | 8 | 3.33 | 27.52 | 25.4 |

**Reviewer#1, Comment #7:** A bus line always has two directions, and the route of a line in different direction may be in different modal. Dose the proposed method considers bus line direction.

**Author response:** Thanks for your valuable comment.

**Author action:** Necessary explanations have been supplemented before model formulation. The proposed method focuses on the peak-hour bus operation optimization in one direction along the corridor considering the following factors.

(1) During the same time period, there appears a great difference in the passenger volumes on two directions. E.g., when the hourly passenger volume of direction reaches the peak point, the volume on the other direction is usually below one-third of the peak volume. Therefore, the bus operation schemes of two directions should be optimized separately, otherwise the bus vehicle capacity on the low-volume direction will be excessively wasted, or the basic level of service on the high-volume direction couldn’t be guaranteed.

(2) The upstream and downstream passenger volumes show a strong distribution heterogeneity with each other. On one hand, the stations along the two directions are not identical to each other, namely the number of stations, the station location, and the distribution of station spacing are different. On the other hand, the high-volume sections on the two directions are different.

Therefore, the bus operation on each running direction can be regarded as an independent optimization problem. Given necessary input data and parameters, our proposed model is feasible to optimize the bus operation on each direction and output different schemes. As to the bus vehicle dispatching at terminal depots or stations, it is another operation decision problem related to the vehicle resource allocation, driver scheduling, vehicle turnover connection and etc., which will be discussed in our future study. Thanks again for your comment.

**Reviewer#1, Comment #8:** The title of this paper is “Collaborative Optimization of Multi-modal Transport Solutions for Urban-Rural Bus Routes”. However, the whole method doesn’t refer the difference between urban-rural bus and urban bus. Compared with urban, what are urban-rural bus characteristics.

**Author response:** Thanks for your comment.

**Author action:** In order to address the difference between urban-rural bus and urban bus, necessary descriptions have been added in the introduction part in revised manuscript as follows.

Compared with the urban bus, urban-rural bus owns the following characteristics:

(1) The average trip distance is longer. Due to the differences in land use and population density, there are only a few passenger distributing stations along the urban-rural corridors, the average trip distance of urban-rural bus is longer than the urban bus. Meanwhile, most stations on the urban-rural corridors have fallen into disuse, leading to the increase of actual stop spacing.

(2) Passenger trips are more concentrated in the peak hour period. Since the urban-rural bus mainly serves the commuting trips along the urban-rural corridor, the passenger volume during rush hours usually account for 50% of daily trips. While the rush hour volume on urban bus usually accounts for 15 to 20% of daily trips, because there still exist large passenger volumes with the purposes of business travel, medical travel, recreation travel, shopping travel, tour travel during the non-peak hours.

(3) The directional volume imbalance is more obvious. For the urban bus corridor, there is no significant difference between the two directional volumes during the same time period. While the tide traffic along the urban-rural corridor leads to an apparent direction volume disequilibrium, e.g., in the morning peak hour of workday, the number of passengers towards the urban area is bigger than the passengers from urban district to urban area.

**Reviewer#1, Comment #9:** Operator service cost is one of optimal objectives. But in practical work, the income of bus line is always an important factor in optimization. Therefore, could author add income in the model.

**Author response:** Thanks for your valuable advice. Among the components of bus income and expense, the vehicle depreciation, driver salary and energy consumption are considered in the proposed optimization model, in order to reduce the variable operation cost to avoid more operating losses. As to the income items of urban-rural bus, government subsidies and advertising revenues differ in the policy supports and negotiation results, and the ticket revenue only accounts for a small proportion in the total income, therefore the ticket revenue is ignored in the objective. The reasons why we didn’t take the income into the objective are listed as follows:

(1) The financial subsidies of different bus routes are hard to determine. In China, the bus systems operated are managed by the government sectors, and both urban bus and urban-rural bus operate as public-welfare transportation services. Many bus routes couldn’t keep operation without financial subsidies. The urban-rural bus faces more economic losses than the urban bus due to its low passenger volume.

(2) Since the development of urban-rural transportation occupies an important position in equalization development, the local government has to provide the urban-rural bus service to guarantee the urban-rural connection, regardless of the fixed vehicle and infrastructure investment, while the bus operator will pay attention to the daily operation cost to control the expenditure.

(3) The income and expense composition of bus service is rather complicated. For a bus operator, the income comes from the government subsidy, advertising revenue, ticket revenue, the expenditure includes the vehicle purchase, vehicle maintenance, vehicle depreciation, driver salary, energy consumption, management fee and etc.

**Author action:** In the revised version, the ticket income has been considered as an auxiliary indicator when selecting the combined scheme of three service modals:

By introducing the ticket income item, the ticket incomes of three bus service modals under representative schemes can be obtained, shown in **Table S3** to **Table S5**. According to current ticket pricing strategy, the all-stop bus and shuttle bus both charge a ticket of 2 Yuan/pax, and the express bus charges under segmented prices of 5 Yuan/pax and 10 Yuan/pax. Note that passengers using preferential tickets or IC cards are ignored here, in order to ensure the comparability among schemes. The total ticket incomes under scheme 2, scheme 5 and scheme 7 are 1710 Yuan, 1567 Yuan and 1753 Yuan, respectively. Despite scheme 7 owns the highest ticket income, its operating cost is also the highest. Therefore, scheme 2 is selected as the recommendation operation scheme.

**Table S3. Ticket income analysis under scheme 2 (Unit: Yuan)**

| Station ID | Total volume | All-stop bus | | Express bus | | Shuttle bus | |
| --- | --- | --- | --- | --- | --- | --- | --- |
|  |  | Volume | Ticket income | Volume | Ticket income | Volume | Ticket income |
| 1 | 50 | 46 | 92 | 4 | 20 | 0 | 0 |
| 2 | 30 | 30 | 60 | 0 | 0 | 0 | 0 |
| 3 | 50 | 50 | 100 | 0 | 0 | 0 | 0 |
| 4 | 36 | 36 | 72 | 0 | 0 | 0 | 0 |
| 5 | 45 | 45 | 90 | 0 | 0 | 0 | 0 |
| 6 | 40 | 26 | 51 | 14 | 72 | 0 | 0 |
| 7 | 52 | 33 | 67 | 19 | 94 | 0 | 0 |
| 8 | 45 | 29 | 58 | 16 | 81 | 0 | 0 |
| 9 | 120 | 49 | 98 | 0 | 0 | 71 | 142 |
| 10 | 88 | 36 | 72 | 21 | 106 | 31 | 62 |
| 11 | 40 | 16 | 33 | 0 | 0 | 24 | 47 |
| 12 | 36 | 15 | 30 | 0 | 0 | 21 | 42 |
| 13 | 35 | 14 | 29 | 0 | 0 | 21 | 41 |
| 14 | 37 | 15 | 30 | 1 | 5 | 21 | 42 |
| 15 | 38 | 38 | 76 | 0 | 0 | 0 | 0 |
| 16 | 0 | 0 | 0 | 0 | 0 | 0 | 0 |
| Sum | 742 | 479 | 957 | 75 | 377 | 188 | 376 |

**Table S4. Ticket income analysis under scheme 5 (Unit: Yuan)**

| Station ID | Total volume | All-stop bus | | Express bus | | Shuttle bus | |
| --- | --- | --- | --- | --- | --- | --- | --- |
|  |  | Volume | Ticket income | Volume | Ticket income | Volume | Ticket income |
| 1 | 50 | 46 | 92 | 4 | 20 | 0 | 0 |
| 2 | 30 | 30 | 60 | 0 | 0 | 0 | 0 |
| 3 | 50 | 50 | 100 | 0 | 0 | 0 | 0 |
| 4 | 36 | 36 | 72 | 0 | 0 | 0 | 0 |
| 5 | 45 | 45 | 90 | 0 | 0 | 0 | 0 |
| 6 | 40 | 40 | 80 | 0 | 0 | 0 | 0 |
| 7 | 52 | 52 | 104 | 0 | 0 | 0 | 0 |
| 8 | 45 | 45 | 90 | 0 | 0 | 0 | 0 |
| 9 | 120 | 49 | 98 | 16 | 78 | 55 | 110 |
| 10 | 88 | 36 | 72 | 0 | 0 | 52 | 104 |
| 11 | 40 | 16 | 33 | 1 | 5 | 23 | 45 |
| 12 | 36 | 15 | 30 | 2 | 10 | 19 | 38 |
| 13 | 35 | 14 | 29 | 3 | 15 | 18 | 35 |
| 14 | 37 | 15 | 30 | 2 | 10 | 20 | 40 |
| 15 | 38 | 38 | 76 | 0 | 0 | 0 | 0 |
| 16 | 0 | 0 | 0 | 0 | 0 | 0 | 0 |
| Sum | 742 | 528 | 1056 | 28 | 138 | 186 | 373 |

**Table S5. Ticket income analysis under scheme 7 (Unit: Yuan)**

| Station ID | Total volume | All-stop bus | | Express bus | | Shuttle bus | |
| --- | --- | --- | --- | --- | --- | --- | --- |
|  |  | Volume | Ticket income | Volume | Ticket income | Volume | Ticket income |
| 1 | 50 | 32 | 64 | 18 | 90 | 0 | 0 |
| 2 | 30 | 30 | 60 | 0 | 0 | 0 | 0 |
| 3 | 50 | 50 | 100 | 0 | 0 | 0 | 0 |
| 4 | 36 | 36 | 72 | 0 | 0 | 0 | 0 |
| 5 | 45 | 29 | 58 | 16 | 81 | 0 | 0 |
| 6 | 40 | 26 | 51 | 14 | 72 | 0 | 0 |
| 7 | 52 | 33 | 67 | 19 | 94 | 0 | 0 |
| 8 | 45 | 29 | 58 | 16 | 81 | 0 | 0 |
| 9 | 120 | 57 | 113 | 4 | 20 | 59 | 119 |
| 10 | 88 | 41 | 83 | 2 | 10 | 45 | 89 |
| 11 | 40 | 19 | 38 | 0 | 0 | 21 | 42 |
| 12 | 36 | 17 | 34 | 0 | 0 | 19 | 38 |
| 13 | 35 | 16 | 33 | 0 | 0 | 19 | 37 |
| 14 | 37 | 17 | 35 | 0 | 0 | 20 | 39 |
| 15 | 38 | 38 | 76 | 0 | 0 | 0 | 0 |
| 16 | 0 | 0 | 0 | 0 | 0 | 0 | 0 |
| Sum | 742 | 470 | 941 | 90 | 448 | 182 | 364 |

**Reviewer#1, Comment #10:** The proposed model contains a lot of parameters. Therefore, the parameter estimation is important and necessary. Please supplement the part in detail.

**Author response:** Thanks for your valuable suggestion. In the revised version, a section of parameter calibration has been supplemented as an independent part.

In the programming model, parameters needing estimation or calibration include the trip volume distribution *q_ij_* among different stations, modal choice ratio, value of time *vot*, running distance *Lij* between stations, operation characteristics of different bus modals. The operation characteristics of different service modals have already illustrated in the original manuscript.

**Author action:** The following description of input parameters have been supplemented in the revised version.

(1) Trip volume distribution

As previously addressed, this paper focus on the bus operation optimization on one direction, please see the response to your 7^th^ comment. (Reviewer #1 Comment#7). The input origin-destination trip matrix between stations along current direction is shown below. As indicated in **Table S6**, the total number of passenger exchanges during the peak hour is 742 pax/h, trips between stations range from 0 to 42, and the station 9 (*Changsheng Station*) owns the highest passenger generation of 120 pax/h, the station 14 (*Xiazhuang Station*) owns the highest passenger attraction of 164 pax/h.

**Table S6. Peak-hour trip distribution among different stations (Unit: pax/h)**

| **O\D** | **1** | **2** | **3** | **4** | **5** | **6** | **7** | **8** | **9** | **10** | **11** | **12** | **13** | **14** | **15** | **16** |
| --- | --- | --- | --- | --- | --- | --- | --- | --- | --- | --- | --- | --- | --- | --- | --- | --- |
| **1** | 0 | 5 | 5 | 10 | 22 | 0 | 0 | 5 | 3 | 0 | 0 | 0 | 0 | 0 | 0 | 0 |
| **2** |  | 0 | 3 | 8 | 8 | 8 | 2 | 1 | 0 | 0 | 0 | 0 | 0 | 0 | 0 | 0 |
| **3** |  |  | 0 | 2 | 4 | 15 | 6 | 8 | 10 | 5 | 0 | 0 | 0 | 0 | 0 | 0 |
| **4** |  |  |  | 0 | 2 | 1 | 12 | 10 | 10 | 1 | 0 | 0 | 0 | 0 | 0 | 0 |
| **5** |  |  |  |  | 0 | 1 | 7 | 18 | 16 | 3 | 0 | 0 | 0 | 0 | 0 | 0 |
| **6** |  |  |  |  |  | 0 | 0 | 8 | 25 | 7 | 0 | 0 | 0 | 0 | 0 | 0 |
| **7** |  |  |  |  |  |  | 0 | 0 | 11 | 34 | 5 | 2 | 0 | 0 | 0 | 0 |
| **8** |  |  |  |  |  |  |  | 0 | 5 | 22 | 5 | 10 | 3 | 0 | 0 | 0 |
| **9** |  |  |  |  |  |  |  |  | 0 | 10 | 3 | 12 | 30 | 40 | 25 | 0 |
| **10** |  |  |  |  |  |  |  |  |  | 0 | 3 | 20 | 20 | 42 | 3 | 0 |
| **11** |  |  |  |  |  |  |  |  |  |  | 0 | 0 | 10 | 30 | 0 | 0 |
| **12** |  |  |  |  |  |  |  |  |  |  |  | 0 | 1 | 32 | 3 | 0 |
| **13** |  |  |  |  |  |  |  |  |  |  |  |  | 0 | 20 | 15 | 0 |
| **14** |  |  |  |  |  |  |  |  |  |  |  |  |  | 0 | 35 | 2 |
| **15** |  |  |  |  |  |  |  |  |  |  |  |  |  |  | 0 | 38 |
| **16** |  |  |  |  |  |  |  |  |  |  |  |  |  |  |  | 0 |

(2) Modal choice ratio at bus stations

Since there are three bus service modals running along the corridor, passengers will choose the corresponding bus modal according to their own expectation and income level. E.g., the rapid bus can save the travel time due to the limited stop frequency and higher travel speed, but its ticket price is much higher, usually 2 to 4 times higher than the all-stop bus and the shuttle bus. Therefore, passengers have to judge and weigh the time-saving benefits and the ticket expenditures when choosing bus modals. Because it is hard to qualify the individual expectation, a survey has been conducted to estimate the choice probability, which was performed together with the individual attribute investigation. The aggregate sample distribution of modal choice is indicated in **Table S7**.

**Table S7. Aggregate modal choice distribution at different sections**

| Parameters | Sections only covered by all-stop and express bus | | Sections covered by all three modals (Shuttle bus running section) | |
| --- | --- | --- | --- | --- |
|  | Number of samples | Choice ratio | Number of samples | Choice ratio |
| All-stop bus choice,  | 26 | 65% | 15 | 41% |
| Express bus choice,  | 14 | 35% | 5 | 13% |
| Shuttle bus choice,  | / | / | 17 | 46% |
| Sum | 40 | 100% | 37 | 100% |

(3) Value of time

Please see our response to your 5^th^ comment. (Reviewer #1, comment #5)

(4) time consumption by stopping at station

As to the time consumption by stopping at station (*t_s_*), the speed loss time caused by decelerating and accelerating near station *s* is about 12 s, the door opening and closing time of bus vehicle is about 5 s, the average boarding and alighting time is about 8 s, therefore the parameter *t_s_* takes the value of 25 s.

**Reviewer#1, Comment #11:** The data description is inadequate. Please describe all data collection in this paper by table and samples.

**Author response:** Thanks for your valuable suggestion.

**Author action:** As revealed in the foregoing responses, necessary Tables and samples have been added in the revised manuscript with proper descriptions.

**Reviewer#1, Comment #12:** A comparison with other model or method should be taken in discussion, which is used to prove your model is better.

**Author response:** Thanks for your valuable advice. The reason why we didn’t perform the comparison with other model or method lie in the following aspects:

(1) Different methods correspond to different scenarios and purposes. On one hand, for the optimization of rural-bus bus operation, some methods focus on the timetable connection optimization at transfer stations in the suburban area, some methods focus on joint optimization of ticket pricing and operation scheme of a certain service modal, and some models emphasizes the adjustment of operation route and stop station. On the other hand, for the optimization of multi-modal bus services, the application scenarios of related studies majorly target at the busses running on city arterials or connecting transportation hubs, which require different demands in punctuality, comfortability and convenience.

(2) Different models have different inputs and constraints. In terms of input parameters, besides the passenger OD trip distribution, station spacing and basic operation parameters, different optimization model has different input parameters. E.g., bus energy consumption calls for additional inputs of route alignment, on-board travel time is estimated according to the section traffic volume and congestion index, and the passengers waiting time needs to be calibrated upon the arrival rate and service choice probability. In terms of model constraints, the limitation varies with the bus service scenario, e.g., the value of minimum section capacity usage and the number of maximum express bus stops are different in different studies.

**Author action:** In view of the foregoing differences, it is hard for us to perform the model or method comparison, and meanwhile, as a planning model, the solving algorithm of the proposed model doesn’t need to be efficient despite our solving procedure is time-saving. However, we have added a comparison between the optimal scheme and the actual scheme in the discussion, as indicated in the **Table S8**. Hope for your understanding, thank you.

**Table S8. Comparison between original scheme and optimized scheme during the morning peak**

| Service modal  Comparison indicator | | All-stop | | express | | shuttle | |
| --- | --- | --- | --- | --- | --- | --- | --- |
|  |  | original | optimal | original | optimal | original | optimal |
| Scheme | Departure frequency (veh/h) | 3 | 4  (33% ↑) | 3 | 1  (67% ↓) | 5 | 4  (20% ↓) |
|  | Average stops | / | / | 7 | 5  (-2) | 8 | 6  (-2) |
| Cost | Operation cost (Yuan) | 381 | 507  (33% ↑) | 124 | 38  (69% ↓) | 214 | 162  (24% ↓) |
|  | Trip time (h) | 121 | 156  (39% ↑) | 38 | 21  (48% ↓) | 53 | 59  (11% ↑) |
| Section  capacity | Supply (pax/h) | 150 | 200  (33% ↑) | 105 | 35  (67% ↓) | 100 | 80  (20% ↓) |
|  | Average usage | 0.57 | 0.59  (3.5% ↑) | 0.41 | 0.54  (31.7% ↑) | 0.72 | 0.76  (5.6% ↑) |

According to the comparison results, the findings can be concluded as:

(1) With the increase of all-stop bus departure frequency, the operation cost and capacity increase synchronously with the same increasing of 33%, while the average section capacity usage only increases by 3.5% under a small increase of passenger attraction.

(2) The decreases in the departure frequency and the average stops of express bus cause a significant passenger volume loss, and the operation cost and trip time decrease accordingly.

(3) The decreases in the departure frequency and the average stops of shuttle bus cause a 24% decrease in the operation cost, while the trip time has increased by 11% due to the additional volume shifted from the express bus.

(4) Generally, under the optimized scheme, the total operation cost decreases from 719 Yuan to 707 Yuan, but the total trip time increases from 212 h to 246 h. From the perspective of vehicle purchasing and maintenance, the benefit of the optimal scheme outperforms the original one. Meanwhile, the average section capacity usage of all three modals gets increased under the optimal scheme.

Meanwhile, the following literatures have been added in the revised discussion section:

1. Takamatsu M, Taguchi A. Bus timetable design to ensure smooth transfers in areas with low-frequency public transportation services. Transportation Science, 2020, 54(5): 1238-1250.
2. Steiner K, Irnich S. Schedule-based integrated intercity bus line planning via branch-and-cut. Transportation Science, 2018, 52(4): 882-897.
3. Liu Y, Yao E, Liu S. Energy consumption optimization model of multi-type bus operating organization based on time-space network. Applied Sciences, 2019, 9(16): 3352.
4. Zhang SJ, Jia SP, Mao BH, Ma CR, Zhang T. Influence of passenger trip distance distribution on competitiveness of bus lines in urban rail transit network. Journal of Zhejiang University(Engineering Science), 2019, 53(2): 292-298.

**Reviewer#2, Comment #1:** Please clearly present the main contributions of this study.

**Author response:** Thanks for your valuable suggestion. The main contributions of this paper have been clearly listed in the conclusion section. The detailed description is shown in our response to Reviewer#1, Comment #1. Thank you.

**Reviewer#2, Comment #2:** For all-stop buses, express buses, and shuttle buses, their competition and cooperation relationships should be considered in the optimization.

**Author response:** Thanks for your valuable suggestion. Their cooperation relationship has been considered in the original optimization, embodied in constraint (10), constraint (11) and constraint (13), for the purposed of improving capacity usage and guaranteeing service quality. As to their competition, the stop frequency for express bus (constraint 14) and the shuttle bus running section constraint (constraint 15) have already been considered, where constraint (14) is used to guarantee the rapidity of express bus, and constraint (15) is used to determine the high-volume sections for shuttle bus operation. However, constraints revealing the competition relationship among bus modals were lacked.

**Author action:** In the revised model, additional constraints on the stop plan of express have been added considering its competition with other two bus modals.

For the stations passed by all-stop and express bus vehicles, the stop plan for the express bus at station should satisfy the constraint below. The first item on the left side represents the number of passengers at station *i* boarding the express bus, only when the express bus stops at station *j* () and the trip volume from station *i* to station *j* exists, station *i* will generate the express bus passengers. The second item on the left side denotes the number of passengers boarding the all-stop bus, and the item on the right side is the aggregate boarding passengers at station *i*.

Similarly, for the stations passed by three bus modal vehicles, the stop plan for the express bus at station *i* should satisfy:

It should be noted that the selection choice of different bus modals are assumed as determined values according to a questionnaire investigation, while by nature they are dynamic at different stations under different passenger trip distance, trip purpose, ticket pricing and comfortability demands, which will be explored in our future studies.

**Reviewer#2, Comment #3:** Case study could be presented with more details, such as the description of the urban-rural bus corridor from Jasmine Ecological Park to West Xiangyang Bridge and a figure of such corridor.

**Author response:** Thanks for your comment. There were a figure describing the corridor in figure 2 behind the original manuscript (**Figure S2**), where Huaisi lizhuang is the bus stop name near the Jasmine Ecological Park.

**Figure S2. The urban-rural corridor and active bus station distribution**

**Author action:** To better describe the corridor, a table of bus routes along the corridor has been added in the revised version (**Table S9**).

**Table S9. Characteristics of representative bus routes along the urban-rural transportation corridor**

| Service model | Bus route | Operation period | Average departure interval (min) | Travel speed  (km/h) | Average stop spacing  (m) |
| --- | --- | --- | --- | --- | --- |
| Suttle bus | 15 | 6:10-18:35 | 14 | 29.8 | 549 |
|  | 15 special | 16:33, 17:23, 18:11 | 49 | 28.5 | 549 |
|  | 105(night) | 18:45-21:40 | 25 | 31.2 | 584 |
| Rapid bus | Youyang sightseeing | 7:20-18:30 | 30 | 35.1 | 2692 |
|  | Songyang express | 5:40-17:30 | 30 | 34.5 | 3701 |
| All-stop bus | 103 | 5:40-17:55 | 19 | 26.1 | 499 |
|  | Guoyang trunk | 6:00-18:40 | 60 | 25.4 | 919 |

Meanwhile, in order to illustrate the bus corridor, more information has been uploaded as part of the raw data in the submission system, as summarized in the table below. Note that the 103 bus line and Songyang trunk line are all-stop bus, while part of their stations are deviated but near current corridor. **Table S10** shows the operation information along the urban-rural corridor.

**Table S10. Bus routes running along the urban-rural corridor**

| **#** | **Station** | **Stop bus route number** | **Platform type** |
| --- | --- | --- | --- |
| 1 | huaisi lizhuang(Jasmine park) | 103, 105（night） | Stop-board |
| 2 | automobile park | 103, 105（night） | Stop-board |
| 3 | baojia village | 103, 3, 105（night） | Stop-board |
| 4 | west baojia village | 103, 105（night） | Stop-board |
| 5 | huaisi hub | 15, 15 special, 103, 105（night） | bus bay |
| 6 | buzhuang | 15, 15 special, 103, 105（night） | bus bay |
| 7 | qiuzhuang | 15, 15 special, 103, 105（night） | bus bay |
| 8 | flower pavilion | 15, 15 special, 103, 105（night） | bus bay |
| 9 | tangzhuang | 15, 15 special, 103, 105（night）, Youyang sightseeing | bus bay |
| 10 | fangxiang | 15, 15 special, Guoyang trunk | bus bay |
| … | … | … | … |
| 31 | jiedao | 315 | Stop-board |
| 32 | taiping village | Songyang express, Guoyang trunk | Stop-board |
| 33 | five-star bridge | Songyang express, Guoyang trunk | Stop-board |
| 34 | wuzhuang estate | Songyang express, Guoyang trunk | Stop-board |
| 35 | huangzhuang | Songyang express, Guoyang trunk | Stop-board |
| 36 | xiazhuang | 8, 309, Youyang sightseeing, Guoyang trunk | Stop-board |
| 37 | songqiao community | 8, 309, Youyang sightseeing, Guoyang trunk | Stop-board |
| 38 | food market | 8, Youyang sightseeing, Guoyang trunk | Stop-board |
| 39 | west xiangyang bridge | Songyang express, Guoyang trunk | Stop-board |

**Reviewer#2, Comment #4:** The language needs more work.

**Author response:** Thanks for your advice.

**Author action:** The authors have improved the language by a tool named “Ginger Writer”, which helps a lot in word using and grammar checking. Please see the revised manuscript.

**Editors, Keypoint #1:** Please remove any funding-related text from the manuscript and let us know how you would like to update your Funding Statement.

**Author response:** Thank you! Funding-related text has been removed.

**Editors, Keypoint #2:** Please confirm at this time whether or not your submission contains all raw data required to replicate the results of your study. Authors must share the “minimal data set” for their submission.

**Author response:** Thank you!

**Author action:** The following files have been uploaded as the raw data set:

1-Current station spacing distance.xlsx

2-Bus corridor information.xlsx

3-Peak-hour passenger volume estimation (all transportation modals).xlsx

4-Reconstructed bus stations.xlsx

5-Peak hour origin-destination volume distribution matrix (bus transit).xlsx

6-Station alighting and boarding passengers.xlsx

7-Lingo codes.docx

**Editors, Keypoint #3:** We note that Figure 2 in your submission contain copyrighted images. We require you to either (1) present written permission from the copyright holder to publish these figures specifically under the CC BY 4.0 license, or (2) remove the figures from your submission.

**Author response:** The photos in Figure 2 are photographed by the authors during the field investigation. The authors approve of the publication of these pictures under the CC BY 4.0 license.
